# The integration of idioms of distress into mental health assessments and interventions: a systematic review

**DOI:** 10.1017/gmh.2019.5

**Published:** 2019-05-07

**Authors:** C. Cork, B. N. Kaiser, R. G. White

**Affiliations:** 1 School of Education, University of Glasgow, 11 Eldon Street, Glasgow G3 6NH, UK; 2 Department of Anthropology, University of California San Diego, La Jolla, CA, USA; 3 Institute of Human and Life Sciences, University of Liverpool, G.10, Whelan Building, Brownlow Hill, Liverpool L69 3GQ, UK

**Keywords:** Assessment, cultural concepts of distress, idioms of distress, intervention, translation

## Abstract

**Background.:**

Psychiatric diagnostic manuals recognise the importance of local expressions of distress in culturally diverse settings [i.e. idioms/cultural concepts of distress (CCDs)], yet there is a lack of consensus on how these should be incorporated into mental health related research.

**Aims.:**

To perform a narrative synthesis and critical review of research exploring how idioms/CCDs have been integrated into assessment measures and interventions.

**Method.:**

A systematic review was conducted in accordance with PRISMA guidelines. An adapted version of the COSMIN checklist was used to assess the quality of the linguistic translation of the idioms/CCDs.

**Results.:**

Twenty-nine papers were included in the final review. Primary qualitative research was the most common method of gathering information about idioms/CCDs. The majority of studies described integrating idioms/CCDs into assessment measures as opposed to interventions. Some studies used information relating to idioms/CCDs to develop novel assessment measures, while others adapted pre-existing assessment measures. The measures generated moderate to high levels of validity. Information relating to the linguistic translation conducted in the completion of the studies tended to be inadequately reported.

**Conclusions.:**

Integrating information about idioms/CCDs into assessment measures can enhance the validity of these assessments. Allocating greater research attention to idioms/CCDs can also promote more equitable exchanges of knowledge about mental health and wellbeing between the Global North and the Global South.

Over the last 10 years, the Global Mental Health (GMH) movement has been engaged in concerted efforts to promote equitable access to mental health services for people worldwide. Commentators, such as Chowdhary *et al*. (2014), have reflected on the importance of culturally and linguistically adapting mental health interventions to optimise engagement with services in local contexts. Researchers from diverse disciplinary fields (including anthropology, psychology and psychiatry) have also advocated for consideration of the local cultural and linguistic contexts when using standardised assessment measures (Mendenhall *et al*., 2016). Nichter (1981, 2010) introduced the term ‘idioms of distress’ to account for ‘socially and culturally resonant means of experiencing and expressing distress in local worlds’ (2010, p. 405). The DSM-5 (American Psychiatric Association, 2013) uses the term ‘cultural concepts of distress’ (CCDs; e.g. *ataques de nervios*, *shenjing shuairuo*, *khyal cap*) as an umbrella term that can be: (1) cultural syndromes, (2) cultural idioms of distress and/or (3) cultural explanations. Many of these documented terms can serve as some or all of these three subgroups of ‘CCDs’. For example ‘khyal cap’ is both an explanatory model – in that it may be perceived as the cause of a set of symptoms – and an idiom of distress – as it is a linguistic term used among particular groups to talk about suffering or distress. A review of epidemiological studies of CCDs by Kohrt *et al*. (2014) found that the terminology used to refer to locally distinct ways of expressing distress was not uniform across papers or research teams. Thus in the current paper, which seeks to examine linguistic idioms, the term ‘idioms/CCDs’ will be used to denote the range of linguistic terms that have been employed to date in an attempt to deal with the apparent ambiguity associated with these terms in the literature.

Mendenhall *et al*. (2016) showed that distress detected through narrative interviews can be missed by standardised scales, elucidating the need for more attention to be paid to the context of distress during assessment. While standardised assessment measures can help to facilitate international communication about distress that transcends cultural differences, local concepts of distress can make a rich contribution to the development of instruments and interventions that are valid in the local context (Mendenhall *et al*., 2016). Kohrt & Hruschka (2010*b*) warn of the consequences of not addressing local psychological frameworks in treatment programmes, such as further pathologising and stigmatising the individual, particularly in the case of trauma healing.

Idioms/CCDs have been utilised in a number of ways in psychiatric research and practice, such as being incorporated into assessment questionnaires (Hinton *et al*., 2015) or integrated into models accounting for the development of mental disorder [e.g. Posttraumatic stress disorder (PTSD) and panic attacks] with the aim of improving the effectiveness of interventions among certain cultural groups (Hinton *et al*., 2008). In terms of clinical use, idioms/CCDs can promote improved communication between patients and clinicians in the clinical encounter (Keys *et al*., 2012); improve communication with clients and reduce stigma associated with distress in humanitarian situations (Kohrt & Hruschka, 2010*a*); and improve the accuracy of assessments of psychosocial functioning or psychopathology (Hinton & Lewis-Fernández, 2010; Hinton *et al*., 2012*b*).

The bulk of GMH-related research continues to be published in English-language journals. As such, research into idioms/CCDs frequently requires processes of translation and/or interpretation but also the negotiation of local and global epistemologies. To date, there has not been a systematic effort to identify and review the existing body of research that has sought to integrate information about idioms/CCDs into mental health assessment measures and/or interventions. The current systematic review sought to remedy this by conducting a narrative synthesis of relevant research studies. In addition, an adapted version of the COSMIN (COnsensus-based Standards for the selection of health status Measurement Instruments) checklist (Mokkink *et al*., 2010) – originally developed to assess the methodological quality of studies on measurement properties of health-related patient reported outcomes – was used to assess the quality of the translation of local language descriptions of idiom/CCD data. The aim of this systematic review is to synthesise information relating to the integration of idioms/CCDs into interventions and/or assessment measures for distress. In particular, the review seeks to address the following questions:

1. How was information about idioms/CCDs gathered?
2. How was the information about the idioms/CCDs integrated into the assessment measure and/or intervention?
3. How was the linguistic translation of idioms/CCDs handled?
4. Did studies assess participants' level of understanding of the measure/intervention incorporating idioms/CCDs, and participants' perception of the relevance of the assessment/intervention (e.g. through piloting)?
5. What were the psychometric properties of the assessment measures that were developed?

As a multitude of terms are used to denote idioms/CCDs, for this review they were defined as any distress concept (emotional, psychological, social, etc.) described in the terms used by the DSM (e.g. variations of: idioms of distress, CCDs, cultural syndrome, culturally bound explanatory model) or a distress concept that is described as being culturally influenced.

## Method

The conduct and reporting of this systematic review was guided by the Preferred Reporting Items for Systematic Reviews and Meta-Analyses (PRISMA) Statement (Moher *et al*., 2009).

### Protocol and registration

Systematic review methods and inclusion criteria were documented in a protocol consistent with the PRISMA-P guidelines, as recommended by Shamseer *et al*. (2015). After initial screening, details of the review protocol were recorded on the PROSPERO (http://www.crd.york.ac.uk/PROSPERO/) database (registration number: CRD42017069244).

### Eligibility criteria

Studies were deemed eligible if they:

1. Contained primary data relating to a mental health assessment or intervention that incorporated information about an idiom/CCD
2. The idioms/CCDs addressed were linguistic (i.e. expressed in the form of language rather than behaviour)
3. Were available in English, French or Spanish
4. Discussed how idioms/CCDs were incorporated into a psychosocial assessment measure or intervention

### Information sources

The databases Scopus, PsychInfo, MEDLINE, Web of Science, Sociological Abstracts, CINAHL and ASSIA were searched with no time period limits. The databases were last searched on 19 October 2018. Databases were searched using terms relating to: idioms/CCDs such as ‘local concepts’; intervention and assessment such as ‘measurement’ and ‘treatment’; the CCDs listed by DSM-5 (American Psychiatric Association, 2013) e.g. ‘kufungisisa’; mental illness/health and linguistic/language-based terms such as ‘conceptual equivalence’. The full search strategy can be obtained by contacting the corresponding author. The reference lists of papers meeting inclusion criteria were hand-searched for other potentially eligible studies. Additionally an expert in the field was contacted for information on potentially eligible papers.

### Study selection

The resulting papers were screened independently by two researchers (C.C. and B.K.). In order to establish coherence of understanding of the inclusion criteria, an initial sample of 10 studies was selected at random for the researchers to screen. They compared their results before carrying out independent screening of all articles. The researchers scanned the abstracts and titles before examining the resulting full texts for eligibility based on the pre-determined criteria. Any disparities between the researchers were resolved by thorough discussion of the inclusion/exclusion criteria.

### Data collection process and data items

An extraction form was developed based on the research questions. Information was extracted by one researcher (C.C.) on the following aspects of each study:

1. Aims of the study
2. Population and location
3. Methods of collection of information on idioms/CCDs
4. Language the research was carried out in
5. Terminology used by researcher(s) to refer to idioms/CCDs
6. Methods/frameworks used to develop the assessment measure, or to integrate idioms/CCDs into existing assessment measures and/or psychosocial/psychological interventions
7. Therapeutic models (e.g. cognitive behavioural therapy, interpersonal therapy) that guided the content of the interventions
8. How translation was conducted (if applicable)
9. Whether idioms/CCDs were compared with DSM/ICD categories
10. Results of piloting/psychometric property testing

#### Assessing quality of translation and interpretation

The ‘cross-cultural validity evaluation’ section of the COSMIN checklist (Mokkink *et al*., 2010) was adapted for use in assessing the translation of idiom/CCD data in each paper. As these guidelines were developed for the translation of assessment measures, adjustments were made for the checklist to be used in relation to translation of idioms/CCDs alone. The COSMIN checklist entails choosing one of four response options regarding the level of quality specific to each item that corresponds to four possible gradings (excellent, good, fair and poor). Only fatal flaws are defined as poor quality to reflect the ‘worst score counts’ system of evaluation (Terwee *et al*., 2012). The full questions and response guidelines are available in a manual on the COSMIN website (http://www.cosmin.nl).

## Results

### Study selection

**Fig. 1.**
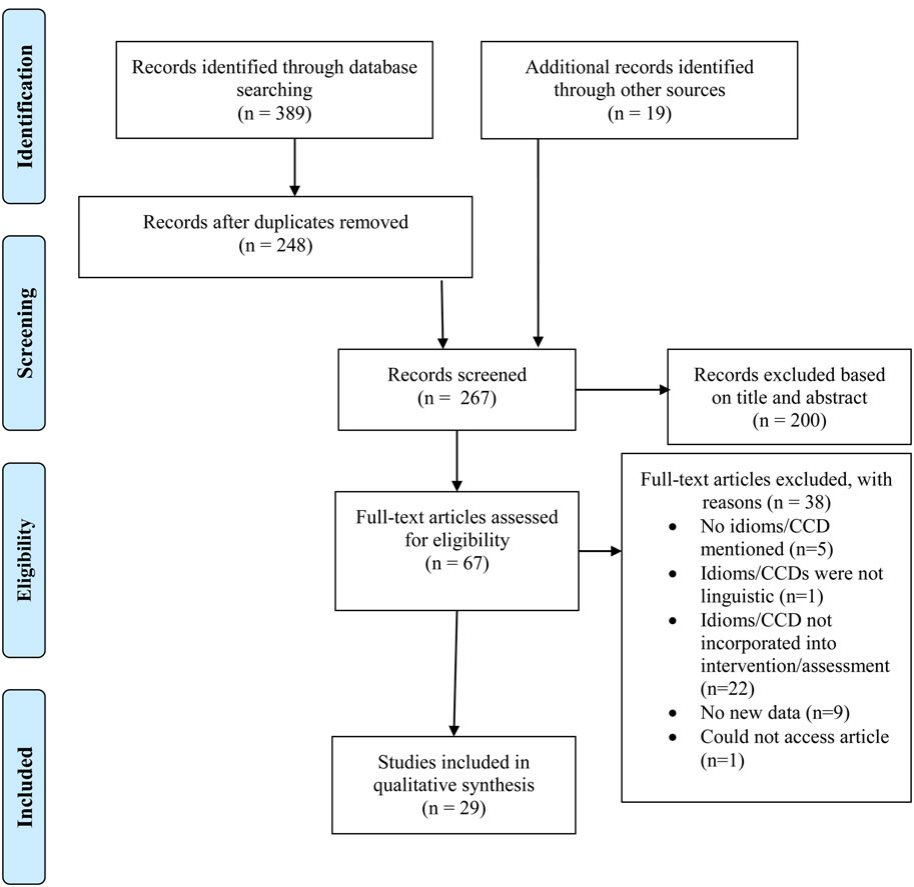
PRISMA flowchart of studies through the screening.

The PRISMA flowchart (see Fig. 1) provides information about the identification and selection of papers. After the full text review, one author (B.K.) included 14 papers that the primary author (C.C.) had not included because of a lack of consensus over the inclusion criterion pertaining to incorporating idioms/CCDs into an assessment/intervention. These differences were resolved after a thorough discussion about the inclusion criteria.

### Study characteristics

Characteristics of included studies are summarised in Table 1. The characteristics are described in the following section based on the 10 aspects extracted as outlined in the Method section. A number of papers discussed the development or use of the same assessment measure. Three discussed the *Acholi Psychosocial Assessment Instrument* (APAI) (Bolton *et al*., 2007; Betancourt *et al*., 2009*a*; Mcmullen *et al*., 2012); two discussed different versions of the *Cambodian Somatic Symptom and Syndrome Inventory* (C-SSI) (Hinton *et al*., 2012*a*, 2013); two discussed the *Kreyòl Distress Idioms* (KDI) inventory (Kaiser *et al*., 2013, 2015); two discussed the *Afghanistan Symptom Checklist* (ASCL) (Miller *et al*., 2006; Rasmussen *et al*., 2014); and two discussed the ‘tension’ scale (Weaver & Hadley, 2011; Weaver, 2017)

**Table 1.** Characteristics of the included studies

	Research objective	Population and location	How was info. gathered on idioms?	How were idioms incorporated?	Label used by researcher	(How) did translation of idioms occur and between what languages?	Were idioms compared with DSM/ICD categories?
Abeyasinghe *et al*. (2012)	Develop and validate a culturally relevant depression scale: the PDS (Peradeniya Depression scale)	Sri Lanka Data collection: 192 case notes from Peradeniya Hospital Validation: 18+; 50 currently depressed outpatients and 50 non-depressed community members.	Clinical notes of Sri Lankan outpatients diagnosed with depression over 10 years examined by a senior consultant psychiatrist based in Peradeniya.	Cultural idioms of distress used to denote symptoms in the PDS	Culturally constructed expression of affect Cultural idioms of distress	Translation did not occur for the scale as it was developed in Sinhalese. However they translated terms into English for the purposes of the paper.	The identified idioms/CCDs were used to contribute to items in a scale which was intended to detect depression.
Bass *et al*. (2008)	To determine if post-partum depression exists in a population in DRC by adapting and validating standard screening instruments	Kinshasa, Democratic Republic of Congo Data collection: 80 mothers living in a peri-urban area who had given birth to a living child in the previous 2 years Validation: 133 women with or without a local depression syndrome	Interviewing a convenience sample of 80 women using qualitative interviewing techniques (i.e. individual free-listing and in-depth interviews with key informants).	Signs and symptoms of the identified local syndrome were added to the questionnaire during adaptation (if they were not already present). Screeners were adapted by using qualitative data terminologies that best reflected the items in the screeners.	Local conceptions of mental illness Local syndrome	All interviewers were bilingual French and Lingala and translated interviews from Lingala to French. Words and concepts that were difficult to translate were discussed amongst interviewers. All interviews were reviewed by study staff. Where the screeners included concepts not reflected in the qualitative data they used standard translation/back-translation methods.	The local syndrome was described as closely approximating the Western model of major depressive disorder.
Betancourt *et al*. (2009*a*)	Evaluate the reliability and validity of the Acholi Psychosocial assessment instrument (APAI)	Validation: 178 War affected adolescents in Northern Uganda with and without the syndromes identified in previous research (Betancourt *et al*., 2009*b*)	For APAI development we are referred to Betancourt *et al*. (2009*b*). Derived from qualitative data from individual free listing and interviews with key informants.	Took the signs and symptoms that comprised each of the five local disorders and the information on local pro social behaviours to generate individual questions and create a subscale for each.	Local syndrome terms	Betancourt *et al*. (2009*b*): authors met with the interviewers to review the interview and transcribe the Luo interview notes into English – stated that they were not professional translators.	Yes local syndromes described as ‘depression-like’ conduct problem etc.
Bolton *et al*. (2007)	To assess effect of locally feasible interventions on depression, anxiety and conduct problem symptoms among adolescent survivors of war and displacement in northern Uganda (using APAI)	314 adolescents (aged 14–17 years) in two camps for internally displaced persons in northern Uganda	See Betancourt *et al*. (2009*a*).	From the problems identified in aforementioned qual. data they chose five judged amenable to intervention. Created an instrument consisting of questions on the symptoms of these five problems. They tested the effects of an intervention on these syndromes.	Local symptom Local syndrome Locally defined syndrome	See Betancourt *et al*. (2009*a*)	They were described as ‘depression-like’ and ‘anxiety-like’. Also said the three local depression problems contain varying (but incomplete) combinations of DSM symptoms of depression and related symptoms.
Choi & Lee (2007)	To develop a culturally tailored nursing programme for patients with Hwa-Byung (HB) and test the effects of the intervention.	Twenty-six employees from Seoul, Korea who stated they had current or past suffering from HB	Previous research studies.	Culturally tailored nursing programme based on traditional processes to vent sorrow/regret (Hahn) called Hahn-Puri. Therapeutic model: Nursing intervention programme consisting mainly of music therapy, drama and group therapy	Culture-bound syndrome Folk illness	It isn't clear whether translation occurred because the idiom used is one already generally recognised (Hwa-Byung). This term was translated for purposes of the paper.	No
Fabian *et al*. (2018)	Develop a culturally appropriate screening tool for mental distress	Maryland County, Liberia Participants aged 19–81 years old from five ethnic groups obtained via maximum variation sampling. Fifty-one participants in interviews, free-lists and pile-sorts. In total, 315 patient charts reviewed from the county mental health clinic.	Mixed methods including free-lists and semi-structured interviews, patient chart reviews, pile-sorts, and focus group discussions.	Focus groups confirmed which terms in each emerging cluster were most appropriate for a screening tool. The most well understood and representative terms became part of the 17 item screening tool.	Idioms of distress	No, carried out in English	No
Fernando (2008)	To develop a measure of psychosocial status that could assess psychosocial functioning in Sinhalese Sri Lankans impacted by traumatic events	Data collection: 20 local informants 25–60 years old. Validation: Sri Lanka 170 Sinhalese (72% women) between 21 and 71 years with differing types of trauma exposure	Individual qualitative interviews with participants involving imagining scenarios where people are experiencing suffering. Idioms gathered from narratives.	From the list of indicators, the most commonly mentioned indicators of distress were used to develop a 27-item measure: the Sri Lankan Index of Psychosocial Status – Adult Version (SLIPSS-A).	Local indicators of distress	Translation did not occur for the scale as the researcher was Sinhalese. Translation occurred for purposes of the paper.	The overall measure was correlated with PTSD but not the itemised idioms/CCDs.
Green *et al*. (2018)	Develop and validate a locally relevant screening tool for perinatal depression	Bungoma, Kenya Data collection: 12 pregnant women/new mothers using maternity services; 38 community health volunteers; 11 mental health professional Validation: 193 pregnant women/new mothers	Free-listing and card sorting in focus groups with both clients and community health volunteers; expert review with health professionals; item analysis by research team	Items from the free-listing and card-sorting exercise were added to the Edinburgh Postnatal Depression Scale and the Patient Health Questionnaire 9. These items consisted of terms that matched previously identified cover terms from commonly used measures, and local constructs that did not match the cover terms.	Local idioms Idioms of distress Local constructs	Kiswahili – English for the purposes of the paper, unclear how.	No, but items were used to detect perinatal depression (DSM-5)
Hinton *et al*. (2011)	To compare a culturally adapted form of CBT compared with applied muscle relaxation (AMR) for PTSD in Latino women	Twenty-four female Latino patients with treatment resistant PTSD. Ten born in Dominican Republic, 14 in Puerto Rico. Location: USA	Previous research studies.	The CA-CBT specifically addresses their idioms of distress. Includes modification of culturally related catastrophic cognitions often related to idioms of distress. Therapeutic model: CBT	Culturally specific syndromes Idioms of distress Cultural syndromes	Idiom/CCD data was not translated.	No
Hinton *et al*. (2012*a*)	To describe a culturally sensitive assessment (C-SSI) and report on the results of a needs assessment	Kampot, Takeo and Kandal provinces Cambodia 139 people identified by members of the community as having signs of continuing psychological distress due to suffering during the Pol Pot period.	A medical anthropologist fluent in Cambodian with experience in Cambodian and South Asian syndromes developed a list of somatic symptoms and cultural syndromes that were found to be of clinical importance in this population.	From this list an initial instrument was piloted and revised based on clinical utility. The abbreviated version contains 12 somatic symptom items and seven syndromes.	Cultural syndromes Culturally emphasised somatic complaints	Cambodian-English for the purposes of the paper. Unclear how.	No
Hinton *et al*. (2013)	To examine the relationship of PTSD to key somatic complaints and cultural syndromes among Cambodians using the CSSI	Massachusetts, USA Assessment: 226 traumatised Cambodian refugees who had presented at psychiatric services.	See Hinton *et al*. (2012*a*)	From this list an initial instrument was piloted and revised based on clinical utility. The full version contains 18 somatic symptoms and 19 syndromes.	Cultural syndromes Culturally emphasised somatic complaints	Cambodian-English for the purposes of the paper. Unclear how.	No
Hinton *et al*. (2018)	To develop an addendum to standard assessments that identifies symptoms and syndromes of clinical importance in a Vietnamese population	Vietnam Data collection: 8 members of the community in Hanoi, 8 adult patients in a psychiatric hospital in central Vietnam. Piloting: Added as part of a major survey of 1004 people in 5 provinces in Vietnam.	List of symptoms and syndromes developed based on: a literature review; clinical experience of the authors in treating anxiety and depression symptoms in Vietnam; and discussions with a Vietnamese psychiatrist on the research team. Participants in two focus groups evaluated this list for relevance, and suggested additional items.	Based on these suggestions a draft version of the Vietnamese Symptom and Cultural Syndrome Addendum (VN SSA) was drafted and piloted with community members and revised to a 35 item Likert type scale. It was then administered as part of a wider survey containing standard Western measures.	Idioms of distress	Vietnamese-English for the purposes of the paper. Unclear how.	No
Ice & Yogo (2005)	Developing and testing the Luo Perceived Stress Scale	Validation: 200 Luo elders Western Kenya (people older than 50, mostly grandparents, half were caregivers of orphans, half were non-caregivers).	A Luo graduate student and an anthropologist familiar with the Luo identified local idioms of distress and emotional well-being that were used to develop the scale.	The scale asked participants if they had experienced any of these identified idioms in the past week	Local idioms of distress Idioms of distress	Prior to the project, the interview was translated (presumably from English) into Dholuo and independently back translated into English. Native Dholuo speakers conducted interviews in local churches and schools.	No
Kaaya *et al*. (2008)	To develop a locally specific screening tool for depression	Dar-es-Salaam region, Tanzania Data collection: 10 key informants and 10 women who had suffered from any of the identified local categories of illness Administration: 787 antenatal clinic attendees	In-depth interviews with key informants to obtain information on symptoms, signs, perceptions (it is assumed of depression). A group of women who had experienced the identified local categories were interviewed on their experiences. From this, 30 different idioms for depressive and anxiety symptoms were identified.	The identified idioms were initially added as a Likert scale to the depression and anxiety subscales of the Hopkins Symptom Checklist–25, and administered alongside the physical and mental health measures of the Short Form Health Survey. From the 47 item HSCL and ethnographic items, logistic regression identified 19 items for the Dar-es-Salaam Symptom Questionnaire (DSQ).	Indigenous expressions Local idioms Locally derived expressions	Kiswahili-English just for the purposes of the paper. Unclear how.	No, but items described as idioms for depressive and anxiety symptoms.
Kaiser *et al*. (2013)	To develop Haitian Kreyol versions of existing instruments. To develop new instruments: the Kreyol Distress Idioms (KDI) and Kreyol Function Assessment (KFA) scales.	Haiti Data collection: 4 people thought to be experiencing mental illness; 31 community leaders and healthcare providers. Community members in 11 focus groups. Pilot testing: 97 rural Haitians randomly sampled in the Central Plateau	Multiple qualitative methods: participant observation, observant participation, interviews and focus group discussions.	Idioms that seem to express mild to moderate mental ill-health were drawn from the qualitative data. These were discussed with two Haitian clinicians and in a focus group discussion with lay community members. Resulting list of 17 items.	Idioms of distress	Developing KDI: Literal and approximate meaning translations of idioms are provided for the purposes of the paper. These idioms were discussed with two Haitian clinicians and in a focus group discussion with lay community members.	No
Kaiser *et al*. (2015)	To evaluate a locally developed screening tool for measuring mental distress in rural Haiti (KDI)	Haiti validation: 408 participants, central plateau residents 18+	Kaiser *et al*. (2013)	Developed a scale (Kaiser *et al*., 2013)	Idioms of distress	Kaiser *et al*. (2013)	States that the idioms of distress appear to be conceptually similar to psychiatric constructs of depression, generalised anxiety, and panic disorder.
Kohrt *et al*. (2016)	To evaluate the validity of the Patient Health Questionnaire (PHQ-9), assess the added value of using idioms of distress, and develop an algorithm for depression detection in primary care	Data collection: 38 18–80 y/o recruited from programme communities. The distribution of caste/ethnicity was representative of the beneficiary population. Validation: Patients 18+ y/o randomly selected from multiple primary care facilities in Chitwan, Nepal.	Previous research documented terms for mental illness which elucidated two categories of distress: heart-mind and brain-mind problems. PHQ cultural translation: During the translation of the PHQ, focus groups evaluated terms used to describe each item and changed some items to include Nepali idioms.	The changes suggested by the groups (including idioms) during translation were incorporated into the PHQ. Patients were also assessed for local idioms of distress. They then developed an algorithm for screening in primary care to optimise detection of depression incorporating the use of local idioms culturally adapted PHQ.	Idioms of distress	The PHQ was translated using van Ommeren's (1999) guidelines from English to Nepali. Translation of heart-mind etc. did not occur for participants.	Idioms were used as indicators for depression in a scale. Brain-mind/heart-mind problems were assessed according to how much they overlapped with CIDI depression screener. The resulting scale was used to detect depression.
McMullen *et al*. (2012)	To measure the prevalence and consider the aetiology of psychological distress in war affected adolescents (APAI)	205 adolescents (12–19) from Gulu, Uganda	See Betancourt *et al*. (2009*a*)	These symptoms were adapted into a Likert scale of frequency (Betancourt *et al*., 2009*a*).	Local mental health syndromes Local syndromes	See Betancourt *et al*. 2009*a*.	Yes local syndromes described as ‘depression-like’ conduct problem etc.
Miller *et al*. (2006)	To develop a culturally-grounded assessment measure in conflict and post-conflict situations	Data collection: Convenience sample of 20 community members (10 men, 10 women) from two districts in Kabul, Afghanistan. Validation: 324 adults from eight districts in Kabul	Qualitative interviews with participants involving imagining scenarios where people are experiencing suffering. Idioms gathered from narratives.	The most commonly mentioned indicators of distress were used to create a scale	Idioms of distress Indigenous items	ASCL was initially constructed in English, then translated into Dari by a bilingual Afghan consultant and back-translated again by a second bilingual consultant. Discrepancies were resolved through discussion with the two consultants.	No
Mumford *et al*. (2005)	Develop and validate a questionnaire for depression and anxiety	Pakistan Validation: 300 (150 men; 150 women) patients in inpatient, outpatient services and rural community settings. An additional 30 patients with anxiety disorders were recruited	Seventy-five psychiatric case notes in Urdu, Punjabi and Pashto with diagnosis of anxiety or depressive disorder resulted in a draft of 37 questions. Clinicians searched another 200 case notes using this questionnaire as a template.	Searches resulted in a 75 item questionnaire which was piloted in the current study	Local idioms of emotional distress Common idioms of psychological distress Expressions of distress Local idioms of distress Cultural expressions of distress	No discussion of translation. Unclear whether idioms gathered in each language were translated for the scales in other languages. Unclear whether or how Punjabi and Pashto idioms were translated into Urdu.	Yes, identified which idioms/CCDs were associated with depression and anxiety symptoms (ICD-10) respectively. They then constructed two subscales based on this.
Patel *et al*. (1997)	To develop an indigenous measure of common mental disorders in the Shona language (Preliminary Shona Symptom Questionnaire, PSSQ)	Zimbabwe Data collection: 110 patients with mental illnesses in primary care. Piloting: 95 in- and outpatients Validation: 302 primary care and traditional medical care attenders	Qualitative interviews that elicited idioms of distress of mental disorder.	Elicited idioms were collated and classified into similar groups forming a 47 item questionnaire. Five bilingual health professionals validity of the items.	Idioms of distress	Translation did not occur for the study but English translations (from Shona) are provided for the reader (not clear how they were translated)	No but describes it as a scale that may be useful in detecting common mental disorders
Phan *et al*. (2004)	Develop and validate the Phan Vietnamese Psychiatric scale	Data collection: 180 Vietnamese refugees and immigrants in Sydney Validation: 185 Vietnamese patients from community mental health service; 98 mentally ill Vietnamese patients from psychoeducation classes.	Reviewed relevant Vietnamese literature and an ethnographic survey among Vietnamese refugees and immigrants living in Sydney. Group and individual qualitative interviews.	The resulting idioms were examined by bilingual health professionals and classified into a set of categories resulting in 95 items grouped into five symptom clusters. A questionnaire was created by adapting the items of three of these categories into Likert scale questions.	Idioms Cultural understandings of psychiatric and emotional distress	A section of one scale was translated into Vietnamese with blind back-translation by two qualified translators. Other scales had been previously translated and validated. Idioms were translated from Vietnamese to English for the researchers' purposes.	Yes items are collated according to common psychiatric disorders such as depression, anxiety, and somatisation
Rasmussen *et al*. (2014)	To compare the validity of the self-reporting questionnaire (SRQ-20) and the Afghan symptom checklist	Validation: 1003 adults (500 men, 503 women) at three sites in Afghanistan	See Miller *et al*. (2006)	See Miller *et al*. (2006)	Idioms of distress	See Miller *et al*. (2006)	See Miller *et al*. (2006)
Rasmussen *et al*. (2015)	Develop and validate a Haitian Creole screening instrument for depression	Haiti Data collection: 13 key informants Developing measure: 105 ptcpts aged 14–75 (75 female 30 male) attending rural health clinics (depressed and non-depressed)	Idioms were gathered from previously conducted ethnographic data.	Key informants asked to sort these items and items derived from translated measures developed in the USA into categories of problems that people complain of, and to name and describe these categories. This led to a cluster of items that were similar to depression. These items made up a scale.	Idioms of distress	See Kaiser *et al*. (2013)	Idioms were used to create a ‘depression symptom inventory’ so in a way yes.
Roberts *et al*. (2006)	To develop a Minnesota Multiphasic Personality Inventory (MMPI)-2 scale designed to assess features of the Korean culture-bound syndrome, Hwa-Byung (HB).	Validation: 726 Korean college students (295 men and 431 women) from Eight Korean universities	No info on the Hwa-Byung idiom gathered	The authors selected items from the MMPI-2 item pool that were potential markers for HB. The American experts used an operational definition of HB based symptoms described in the clinical literature. The Korean psychiatrists' item selection was guided by their expertise with HB.	Culture-bound syndrome	Translation of the target idiom (Hwa-Byung) only occurred for the purpose of the paper.	The authors concluded that HB constitutes a combination of depression and somatisation according to DSM-III
Silove *et al*. (2009)	To identify a culturally relevant descriptor of explosive anger and examine community wide prevalence and its associations with past persecution and socio-demographic factors.	Epidemiological study: 1544 Adults, 18 years and older, living in two sucos or villages, one in a rural area, and the other in Dili, Timor-Leste. Qualitative inquiry: 27 community members.	A process of community consultation involving translators, indigenous field personnel and community members.	Prior piloting showed that several of the terms used in an explosive anger questionnaire were not easily translatable into Tetun. They identified potential descriptor terms to be used as items in the questionnaire.	Indigenous descriptor Culturally relevant descriptor	Only occurred for the purposes of the paper	No
Snodgrass *et al*. (2017)	To assess the emotional health and wellbeing of indigenous Sahariya in an approach incorporating both emic and etic elements	Rajasthan and Madhya Pradesh, India Data collection: 25 members of one village (free-list); 60 people from two villages (Likert scale). Survey: 159 heads of households from a relocated village and a buffer zone village. 106 women; 113 men	Initial ethnographic observations and interviews within villages. Free-list interview conducted in one village to elicit relevant positive and negative emotion terms. This list was collapsed into categories during interviews in other villages.	The 40 resulting terms formed the Indian Adivasi positive/negative affect scale, in the form of 5 point Likert responses relating to frequency experienced.	Local emotional experience Idioms of distress Local wellness idioms	Hindi – English for the purposes of the paper. Unclear how.	No
Weaver (2017)	To develop a locally derived definition of tension and a scale to quantify its presence	New Delhi, India Data collection: 62 diabetic and nondiabetic women recruited from 14 public and private clinics. Half had Type 2 diabetes. Pilot: 30 women with diabetes. Interviews: 280 women recruited from clinics, 184 with diabetes.	Freelist interviews in which participants were asked to note down as many symptoms or characteristics of ‘tension’ as they could.	From the most frequently mentioned items a list was created. This was then reviewed item by item by laypeople and mental health professionals. This resulted in a 14 item list which was converted into a tension measurement scale.	Idiom of distress	Hindi-English The idiom ‘tension’ as it is used in Hindi was described for the purposes of the paper (its meaning in context is distinct from English usage)	No, they state that their results suggest that ‘tension’ is related to depression and anxiety but is not equivalent to either.
Weaver & Hadley (2011)	Research connections between type 2 diabetes, mental health, and normative social roles among women living in Delhi, India	New Delhi, India Data collection: 62 diabetic and nondiabetic women recruited from public and private clinics Questionnaire Pilot: 30 diabetic women	See Weaver (2017) Also noted that the use of this idiom was documented in previous studies.	See Weaver (2017)	Explanatory models of depression Idioms of distress	See Weaver (2017)	They state that ‘tension’ resembles depression but does not directly express it.

The studies were conducted in a wide range of locations, with three studies each from Uganda, India, USA and Haiti, two each from Afghanistan, Kenya and Sri Lanka and one each from Cambodia, the Democratic Republic of Congo, Liberia, Vietnam, Tanzania, South Korea, Pakistan, Zimbabwe, Australia, Timor-Leste and Nepal. Four of the 29 studies were conducted with migrant populations in countries other than their country of origin (Phan *et al*., 2004; Roberts *et al*., 2006; Hinton *et al*., 2011, 2013).

#### Identification of idioms/CCDs

Identification of idioms/CCDs was described as being carried out in a number of ways: conducting qualitative interviews as part of the research activity reported in the papers (Patel *et al*., 1997; Miller *et al*., 2006; Bolton *et al*., 2007; Bass *et al*., 2008; Fernando, 2008; Kaaya *et al*., 2008; Silove *et al*., 2009; Betancourt *et al*., 2009*a*; Mcmullen *et al*., 2012; Rasmussen *et al*., 2014; Snodgrass *et al*., 2017; Green *et al*., 2018); utilising qualitative interview data from other research teams (Rasmussen *et al*., 2015); extracting data from clinical case notes (Mumford *et al*., 2005; Abeyasinghe *et al*., 2012) and combined approaches such as reviewing relevant literature as well as gathering qualitative data (Phan *et al*., 2004; Kohrt *et al*., 2016); reviewing literature as well as using information from clinical experience (Hinton *et al*., 2018); participant observation, observant participation and qualitative interviews (Kaiser *et al*., 2013, 2015); and patient chart notes as well as qualitative interviews (Fabian *et al*., 2018). Amongst the studies that used qualitative research methods, the most common ways of gathering information on idioms/CCDs were carrying out free-list interviews, focus groups and in-depth interviews with key informants. These activities were mainly carried out with community samples as opposed to patient samples. Two studies did not discuss how the idioms/CCDs were identified, as they were previously identified in the literature (Choi & Lee, 2007; Hinton *et al*., 2011), while in one paper the methods used to identify idioms/CCDs were not described (Ice & Yogo, 2005). In two papers which described the development of the same assessment, a list of idioms/CCDs was developed by an expert in the field (Hinton *et al*., 2012*a*, 2013). Additional information was gathered on previously identified idioms/CCDs in three studies, two by conducting qualitative research with the sample (Weaver & Hadley, 2011; Weaver, 2017), and the other through rational selection from the Minnesota Multiphasic Personality Inventory (MMPI-2) item pool (Roberts *et al*., 2006).

#### Research language

The papers varied in how they dealt with the language in which the research was carried out. While one paper stated that research was carried out in participants' ‘first language’ (Fernando, 2008), it was not clear whether this was the case with other papers. Research was carried out: with participants who were ‘fluent in’ or ‘spoke the language’ used (Phan *et al*., 2004; Abeyasinghe *et al*., 2012), in the ‘local language’ (Bass *et al*., 2008), in the language of the target ethnic group (Ice & Yogo, 2005; Bolton *et al*., 2007; Betancourt *et al*., 2009*a*; Mcmullen *et al*., 2012); in the preferred language of communication (Hinton *et al*., 2011; Weaver & Hadley, 2011; Rasmussen *et al*., 2014; Weaver, 2017); the most widely spoken language (Patel *et al*., 1997; Kaiser *et al*., 2013, 2015; Rasmussen *et al*., 2015); the national language (Roberts *et al*., 2006; Kaaya *et al*., 2008; Hinton *et al*., 2012*a*, 2013, 2018; Kohrt *et al*., 2016; Green *et al*., 2018) and the more common language in a multi-lingual context (Mumford *et al*., 2005; Miller *et al*., 2006; Snodgrass *et al*., 2017; Fabian *et al*., 2018). In two studies, there was no discussion of what language the research was carried out in (Choi & Lee, 2007; Silove *et al*., 2009).

#### Terminology used to capture idioms/CCDs

Up to 19 different terms were used to allude to the concept of idioms/CCDs, with no single term consistently used across papers and researchers. A range of different terms were employed by the studies to describe idioms/CCDs. Fourteen studies used the term ‘idiom(s) of distress’ (Patel *et al*., 1997; Ice & Yogo, 2005; Mumford *et al*., 2005; Miller *et al*., 2006; Hinton *et al*., 2011; Weaver & Hadley, 2011; Kaiser *et al*., 2013, 2015; Rasmussen *et al*., 2014; Kohrt *et al*., 2016; Snodgrass *et al*., 2017; Weaver, 2017; Green *et al*., 2018; Fabian *et al*., 2018); two studies used the term ‘local idioms of distress’ (Ice & Yogo, 2005; Mumford *et al*., 2005); four studies used the term ‘local syndrome’ (Bolton *et al*., 2007; Bass *et al*., 2008; Betancourt *et al*., 2009*a*; Mcmullen *et al*., 2012); and two studies used the term ‘culture-bound syndrome’ (Roberts *et al*., 2006; Choi & Lee, 2007). See Table 1 for full list of terms used across papers.

#### Integration of idioms/CCDs

##### Assessment measures

Sixteen studies discussed incorporating idioms/CCDs into the items of an assessment measure (including measures made up of a range of idioms). Most discussed developing novel assessments for the same cultural group from whom the idiom/CCDs data were gathered (Patel *et al*., 1997; Phan *et al*., 2004; Ice & Yogo, 2005; Mumford *et al*., 2005; Miller *et al*., 2006; Bolton *et al*., 2007; Fernando, 2008; Betancourt *et al*., 2009*a*; Abeyasinghe *et al*., 2012; Mcmullen *et al*., 2012; Hinton *et al*., 2012*a*, 2013, 2018; Kaiser *et al*., 2013, 2015; Rasmussen *et al*., 2014; Rasmussen *et al*., 2015; Snodgrass *et al*., 2017; Fabian *et al*., 2018); while five discussed the use of idioms/CCDs in the adaptation of pre-existing instruments created elsewhere (Bass *et al*., 2008; Kaaya *et al*., 2008; Silove *et al*., 2009; Kohrt *et al*., 2016; Green *et al*., 2018). Three studies described the development of a checklist or scale for assessing levels of symptoms of a particular idiom/CCD previously identified in the literature: Weaver & Hadley (2011) and Weaver (2017) describe the development of an original checklist within the target population; and Roberts *et al*. (2006) adapted a scale previously created elsewhere for this purpose.

##### Interventions

Two studies discussed the use of idioms/CCDs in an intervention. One discussed the incorporation of idioms/CCDs into an intervention using a cognitive behavioural therapy (CBT) framework (Hinton *et al*., 2011) through specifically addressing *nervios* and *ataque de nervios* during sessions on modifying catastrophic cognitions. The other utilised a programme of music therapy, drama and group therapy based on *Hahn-puri*, or processes that help to vent *Hahn* (sorrow/regret) a concept closely related to *Hwa-Byung* (Choi & Lee, 2007).

#### Translation: COSMIN checklist

**Fig. 2.**
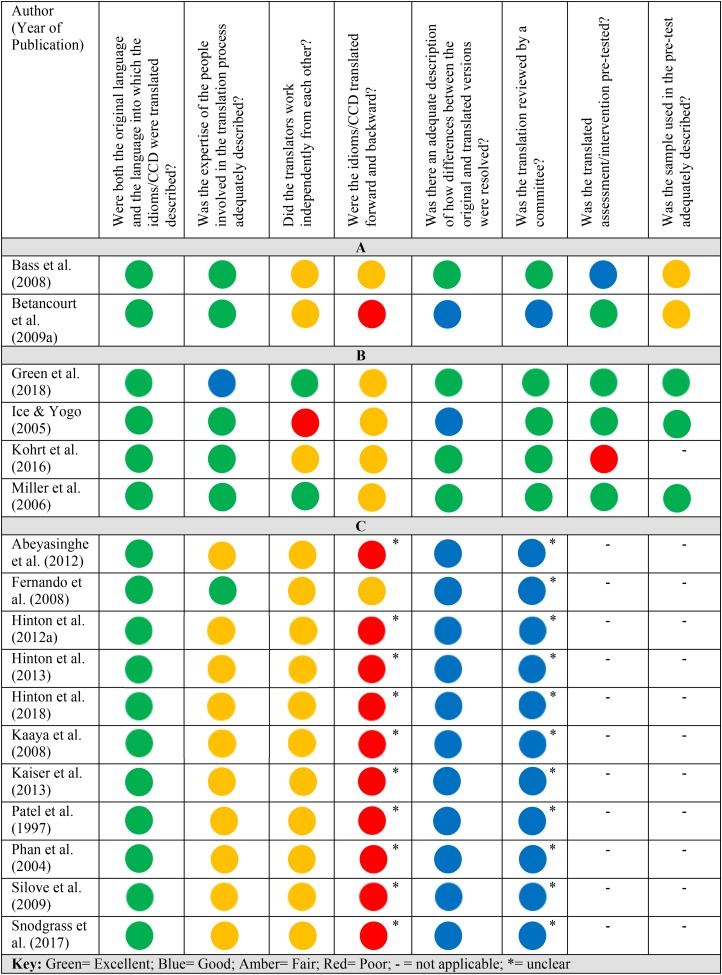
COSMIN evaluation of translation of idioms/CCDs.

The adapted COSMIN checklist was used to assess the quality of the translation of idiom/CCD data in each paper. Four papers neither discussed translation processes of the idiom data, nor referred the reader to another paper that did (Mumford *et al*., 2005; Roberts *et al*., 2006; Choi & Lee, 2007; Hinton *et al*., 2011). However, in all of these, translation of idiom/CCD terms did not occur during the conduct of the research and only occurred for the purpose of writing the paper in English. In two studies, the idiom/CCD (‘tension') was described in terms of how it is used and applied in the local context and thus translation did not occur (Weaver & Hadley, 2011; Weaver, 2017). One study was carried out in English (Fabian *et al*., 2018). Five papers referred the reader to other included papers that discussed the translation of idiom/CCD data (Bolton *et al*., 2007; Mcmullen *et al*., 2012; Rasmussen *et al*., 2014; Kaiser *et al*., 2015; Rasmussen *et al*., 2015). Seventeen papers included in the current review provided information about the translation of idiom/CCD data into English (see Fig. 2).

In Fig. 2 the papers are grouped according to whether the linguistic translation of the idiom/CCD occurred: (A) before the assessment items/intervention content were generated [e.g. in Bass *et al*. (2008), interviewers translated the qualitative interview data from Lingala into French straight after collecting the data]; (B) after the assessment items/intervention content were generated to check the appropriateness of the measure [e.g. in Kohrt *et al*. (2016) a translation of a previously developed instrument was back-translated after the addition of idioms to check its accuracy] and (C) only for the purpose of writing the paper which was published in another language [e.g. in Abeyasinghe *et al*. (2012) the measure development was carried out in Sinhalese, but some idioms are translated and explained for the reader of the article, which is written in English]. It was not appropriate to complete the final two questions on the adapted COSMIN checklist (which pertain to the piloting of a translated assessment measure) for studies in category (C), as translation in these studies only occurred for the purposes of the write-up. The COSMIN checklist works by assigning a rating (excellent, good, fair or poor) partly according to how essential an item is to ensure quality of translation. Therefore some items for some studies may be marked in blue for ‘good’ even if the information for that item was not provided, as it is not deemed to be a fatal flaw.

All papers that discussed the issue of language sufficiently described both the original language and the language into which the idioms/CCDs were being translated. In all studies in categories (A) and (B), the expertise of the translators was adequately described. In papers in category (C), all except for one (Fernando, 2008) did not describe the expertise of the translators. In all papers except for two (Miller *et al*., 2006; Green *et al*., 2018), translators either did not work independently from each other, or it was unclear whether or not they did so. To receive an ‘excellent’ rating on backward and forward translation processes, papers were required to indicate that multiple forward and multiple backward translations were undertaken however, papers variously indicated one forward and one backward translation; only a forward translation; or failed to clearly indicate how it was done. As such, no papers received an ‘excellent’ rating on this section. Four papers adequately described how differences in translations between translators were resolved (Miller *et al*., 2006; Bass *et al*., 2008; Kohrt *et al*., 2016; Green *et al*., 2018), while all remaining papers either described these processes poorly or not at all. In a similar number of studies, the translated idioms/CCDs were reviewed by a committee (Ice & Yogo, 2005; Miller *et al*., 2006; Bass *et al*., 2008; Kohrt *et al*., 2016; Green *et al*., 2018), while in all others there was no review.

Regarding the adapted COSMIN criteria relating to pre-testing assessment measures containing idioms/CCDs, four of the six studies to which this question was relevant pre-tested the measure in the targeted population (Ice & Yogo, 2005; Miller *et al*., 2006; Betancourt *et al*., 2009*a*; Green *et al*., 2018); in one study the measure was pre-tested, but it was unclear whether this was carried out in the target population (Bass *et al*., 2008); while in another study the assessment measure was not pre-tested because the paper was reporting on the development of the measure itself (Kohrt *et al*., 2016). Of the five studies that carried out pre-testing, three adequately described the sample used (Ice & Yogo, 2005; Miller *et al*., 2006; Green *et al*., 2018), while the other two did not (Bass *et al*., 2008; Betancourt *et al*., 2009*a*). Studies in category (C) had poorer quality translation processes than those in categories (A) and (B), with only one paper receiving an ‘excellent’ rating in more than one category (Fernando, 2008).

#### Comparisons with psychiatric diagnoses (DSM/ICD)

Seven studies drew parallels between idioms/CCDs and DSM categories such as: using the idioms/CCDs to contribute to a scale for detecting DSM/ICD psychiatric disorders (Mumford *et al*., 2005; Abeyasinghe *et al*., 2012; Rasmussen *et al*., 2015; Kohrt *et al*., 2016; Green *et al*., 2018); describing them as closely approximating DSM categories (Bass *et al*., 2008); and describing the locally identified problems as containing varying combinations of DSM-IV symptoms of depression and related symptoms (Bolton *et al*., 2007).

In seven studies, comparisons were made between idioms/CCDs and more general psychiatric constructs as opposed to comparing them with specific diagnoses listed in the manuals, i.e. describing idiom/CCDs as: ‘depression-like’ (Bolton *et al*., 2007; Betancourt *et al*., 2009*a*; Mcmullen *et al*., 2012); conceptually similar to psychiatric constructs (Kaiser *et al*., 2015); being representative of a number of psychiatric constructs (Roberts *et al*., 2006); as idioms for depressive or anxiety symptoms (Kaaya *et al*., 2008); or grouped idiom/CCD data according to psychiatric constructs (Phan *et al*., 2004). These comparisons are summarised in Table 1.

#### Efficacy, acceptability and psychometric properties

##### Assessments

All studies that discussed the incorporation of idioms/CCDs into assessment measures sought feedback from relevant stakeholders by piloting the assessment measures prior to use, carrying out focus group discussions during development, or conducting qualitative interventions on the suitability of the language used.

A number of studies included in the review investigated the psychometric properties of the measures that incorporated the idioms/CCDs, including assessing internal, inter-rater and test–retest reliability and content, convergent, discriminant and construct validity^†^^1^. These findings are summarised in Table 2. The majority of studies reported that assessment measures had good psychometric properties. The following studies reported assessment measures that had inadequate psychometric properties: Betancourt *et al*. (2009*a*) reported that the *conduct*, *anxiety* and *prosocial* scales of the Acholi Psychosocial Assessment Instrument (APAI) demonstrated inadequate levels of reliability and validity; in Bass *et al*. (2008), the adapted Hopkins Symptom Checklist (HSCL) and Edinburgh Post-Natal Depression Scale (EPDS) demonstrated only adequate test–retest reliability; in Ice & Yogo (2005), factor analysis showed that the Luo Perceived Stress Scale (LPSS) did not demonstrate a uni-factorial structure; in Mumford *et al*. (2005), specificity of the ‘D’ (depression) scale in a sample of depressive *v*. anxiety patients was not high (64%); in Rasmussen *et al*. (2015) the Zanmi Lasante Depression Symptom Inventory (ZLDSI) had acceptable sensitivity but did less well with specificity, which could not be improved without unacceptable losses in sensitivity.

**Table 2.** Psychometric properties of assessments

Author(s) (year)	Assessment measure	Administration mode	Psychometric testing results
Abeyasinghe *et al*. (2012)	**Peradeniya Depression Scale**	Interviewer administered	Internal consistency: N/A Validity: N/A Inter-rater reliability: N/A Test–retest reliability: N/A Other: The area under the ROC curve was 0.95 (95% CI 0.91–0.99) meaning the PDS can be considered highly accurate. The highest sensitivity and specificity were 87.5% and 88% respectively.
Bass *et al*. (2008)	**Adapted Hopkins Symptom Checklist (HSCL) and Edinburgh Post-partum Depression Scale (EPDS); Screener containing locally sourced items**	Interviewer administered	Internal consistency (*α*): All scales and the composite scale showed good internal consistency – the adapted HSCL, EPDS screener and local screener were 0.86, 0.76 and 0.88 respectively. For the mental health symptoms scale (i.e. HSCL + EPDS) *α* = 0.92. All scales showed good specificity and sensitivity: area under the curve for the detection of the local depression-like syndrome ranged from 0.83 to 0.87, depending on the scale used. The optimal cut-off scores for each scale were all at 80% or greater, except for the specificity of the EPDS cut-off. Validity: Convergence between the mental health symptoms instrument and a locally developed function scale showed evidence of convergent validity [0.34 (*p* = 0.0001)]. There was a consistent increase in depression severity scores with each incremental increase in dysfunction which provides additional evidence of convergent validity for these measures. Discriminant validity testing showed that the mean for cases, 34.9 points (s.e. 1.8), and non-cases, 16.9 points (s.e. 2.4) (identified by Key Informants and self-identification), were substantially and statistically significantly different (*p* < 0.0001). Inter-rater reliability: N/A Test–retest reliability tests showed adequate reliability: The correlations between the scale scores from first interview and the re-interview were: 0.59 (*p* < 0.001), 0.53 (*p* = 0.003), and 0.42 (*p* = 0.02), for the HSCL, EPDS and total scores respectively. Other: N/A
Betancourt *et al*., (2009*a*, *b*)	**Acholi Psychosocial Assessment Instrument (APAI)** Subscales: Ma lwor (anxiety); Kwo Maraco (conduct); Pro social; Depression (combination of two tam, kumu and par)	Interviewer administered	Internal consistency (*α*) For the scales and combinations of scales was adequate or strong: Two tam = 0.87; Kumu = 0.87; Par = 0.84; Ma lwor(anxiety) = 0.70; Kwo Maraco (conduct) = 0.83; Pro social = 0.70; Total depression (combination of two tam, kumu and par) = 0.93; Total APAI problems = 0.93 Validity Concurrent validity – significant mean differences across case status confirmed for all three depression-like syndromes: Case/Non-case Mean(s.d.): Two tam 21.56(8.06)/15.76(8.29); Kumu 16.52 (7.15)/9.33(6.67); Par 17.24(7.69)/11.91(7.08) The mean scores for the corresponding scale scores of ‘cases' of the anxiety syndrome [ma lwor 10.35(5.61)/9.97(5.92)] and the conduct problem syndrome [kwo maraco 5.46(6.48)/2.45(3.09)] were not significantly different from ‘noncases’ as identified by adolescents and caregivers. Inter-rater reliability (*r*) Good for all the APAI depression like problem scales (Two tam = 0.86; Par = 0.78; Kumu = 0.92) Inter-rater reliability was less strong for the anxiety problem scale (ma lwor) (0.62) Poor for the conduct problem scale (kwo maraco) and the prosocial scale (0.25 and 0.35 respectively). Test–retest reliability (*r*) Good for all the APAI depression like problem scales (two tam = 0.79; Par = 0.79; Kumu = 0.89) Less strong for the anxiety problem scale (0.68)
Bolton *et al*. (2007)	**APAI** Depression symptom scale (combination of two tam, kumu and par)	Interviewer administered	Internal consistency (*α*) In adolescents was 0.92 Validity: Concurrent validity was confirmed by substantial and significantly higher scores on the APAI for cases [mean (s.d.)] [45.3, (13.6)] compared with non-cases [15.6, (11.2)], as identified by caregivers and adolescents. Inter-rater reliability: N/A Test–retest reliability (*r*) for the depression symptom scale was 0.84. Other: N/A
Fernando (2008)	**Sri Lankan Index of Psychosocial Status Adult Version (SLIPSS-A)**	Self-report	Internal consistency was high (*α* = 0.92). Validity Content validity was assessed by reviewing the SLIPSS-A items for consistency with the narrative data. 7/12 most frequently endorsed SLIPSS-A items were the most frequently mentioned in narratives. Convergent validity: scores on the SLIPSS-A were significantly correlated with scores on the PCL-C, *r*(99) = 0.75, *p* < 0.000. Predictive validity: the model successfully distinguished between those who had not been exposed to the tsunami and those who had, even after controlling for sample type, χ^2^(3) = 28.7, *p* < 0.000. This was repeated for the community sample alone, and again it significantly predicted trauma exposure χ^2^(1) = 7.72, *p* < 0.005. The model demonstrated adequate ability to predict correctly participants' trauma exposure status, with an overall prediction success rate of 61.6%. Predictive validity: The scores on the SLIPSS-A and an item assessing life satisfaction were strongly negatively correlated, *r*(132) = 0.51, *p* < 0.000. Inter-rater reliability: N/A Test–retest reliability: N/A Other: The western developed PCL-C could not distinguish between trauma groups.
Green *et al*. (2018)	**Perinatal Depression Screening (PDEPS)**, blending EPDS, Patient Health Questionnaire (PHQ-9) items with locally developed items	Interviewer administered	Internal consistency: *α* = 0.81 Validity: Discriminant validity: mean PDEPS score was twice as large for DSM cases than non-cases based on SCID-5-RV diagnosis (13.6 *v*. 6.3) and for cases based on ‘local’ diagnosis (In your clinical judgement, do you think that this woman is ‘depressed’?; 13.3 *v*. 6.2). Convergent validity: Correlation with counsellor rated social and occupational functioning scale (SOFAS) = −0.32 and with self-report rating of wellbeing = −0.25. Construct validity: associated with wealth (*aβ* = −2.7) and work (*aβ* = 2.3) Inter-rater reliability: N/A Test–retest reliability: *r* = 0.62 (enumerator) and *r* = 0.36 (automated phone) Other: Compared with SCID-5-RV diagnosis: sensitivity = 0.90, specificity = 0.90, AUC = 0.89, LR+ = 8.62, LR− = 0.11. Compared with ‘local’ diagnosis: sensitivity = 0.58, specificity = 0.88, AUC = 0.86, LR+ = 5.00, LR− = 0.47. PDEPS outperformed the full PHQ-9 and EPDS in terms of classification accuracy (0.90 *v*. 0.72 and 0.73, respectively).
Hinton *et al*. (2012)	**Cambodian Somatic Symptom and Syndrome** **Inventory SSI (abbreviated)**	Interviewer administered	Internal consistency: N/A Validity: Convergent validity: Correlation with PTSD Check-list (PCL), *r* = 0.69; Harvard Trauma Questionnaire (HTQ), *r* = 0.51; Short Form Health Survey–3 (SF-3), *r* = 0.49 Discriminant validity: Symptom severity for all SSI items increases with increasing PTSD severity. Inter-rater reliability: N/A Test–retest reliability: N/A Other: SSI was more strongly correlated than PCL with HTQ and SF-3.
Hinton *et al*. (2013)	**Cambodian SSI (full)**	Interviewer administered	Internal consistency: Somatic scale, *α* = 0.91; syndrome scale, *α* = 0.88; multi-item syndrome subscales, all *α*s >0.84 Validity: Convergent validity: Correlation with PTSD Check-list (PCL), CSSI total, *r* = 0.67, CSSI somatic scale, *r* = 0.71, CSSI syndrome scale, *r* = 0.63; Short Form Health Survey–12 (SF-12), *r* = 0.7 Discriminant validity: Mean (s.d.) = 2.0 (0.8) for PTSD group and 0.6 (0.5) for non-PTSD group. Symptom severity for CSSI total and both CSSI scales increase with increasing PTSD severity. Inter-rater reliability: N/A Test–retest reliability: N/A Other: SSI was more strongly correlated than PCL with SF-12.
Hinton *et al*. (2018)	**Vietnamese Symptom and Cultural Symptom** **Addendum (VN SSA)**	Interviewer administered	Internal consistency: N/A Validity: Convergent validity: All items were correlated with standardised and summed scale combining Generalised Anxiety Disorder-7 (GAD-7), Posttraumatic Diagnostic Scale (PDS), Patient Health Questionnaire-9 (PHQ-9), *r* = 0.15 to 0.54 Inter-rater reliability: N/A Test–retest reliability: N/A Other: N/A
Ice & Yogo (2005)	**Luo Perceived Stress Scale (LPSS)**	Interviewer administered	Internal consistency: *α* = 0.75 Validity Criterion validity: caregiving, social networks, depression, and cortisol were all associated with LPSS as predicted with the exception of caregiving. Known group validity was examined through comparisons of caregiving groups, genders, marital status, and participation in social groups. While they were generally associated with LPSS in the predicted direction, factor analysis suggested that the LPSS did not represent a single domain. The LPSS requires additional development. Inter-rater reliability: N/A Test–retest reliability: N/A Other: N/A
Kaaya *et al*. (2008)	**Dar-es-Salaam Symptom Questionnaire (DSQ)**	Interviewer administered	Internal consistency: *α* = 0.84 Validity: Construct validity: Items loaded as expected for depression and anxiety symptoms in principal components analysis. Convergent validity: Correlation with Short-Form Health Survey-36 (SF-36), *r* = −0.57 to −0.37 Criterion validity: Significant predictors were economic provisions, control over decisions on household matters, marital status, and education (*β* = −0.21 to −0.09). Inter-rater reliability: *r* = 0.89 Test–retest reliability: *r* = 0.82 Other: N/A
Kaiser *et al*. (2013)	**Kreyòl Distress Idioms (KDI)**	Interviewer administered	Internal consistency: [Mean(s.d.)]: 17.02 (9.6) *α* = .89 Validity: N/A Inter-rater reliability: N/A Test–retest reliability: N/A Other: N/A
Kaiser *et al*. (2015)	**Kreyòl Distress Idioms (KDI)**	Interviewer administered	Internal consistency of the KDI was high (*α* = 0.86). Validity Correlations with other scales – BAI (*r* = 0.67) and BDI (*r* = 0.52) – support convergent and content validity. External validity confirmed by correlation with known risk factors: Number of traumatic events experienced and having a household member with mental distress were both statistically significantly associated with higher KDI score (*aβ* = 1.5 and 5.8, respectively; *p* ⩽ 0.003). Two perceived causes of mental distress were associated with KDI score: those endorsing that relationships can cause mental distress scored on average 3.2 points lower (*p* < 0.001), while those stating that disasters can cause mental distress scored 3.68 points higher (*p* < 0.001). Inter-rater reliability: N/A Test–retest reliability: N/A Other: N/A
Kohrt *et al*. (2016)	**PHQ-9; heart-mind/brain-mind**	Interviewer administered	The internal consistency (*α*) for the PHQ-9 was 0.84. Validity: Validated by comparing the researcher administrated Nepali PHQ-9 and the CIDI. The CIDI and PHQ-9 were compared identifying an area under the curve (AUC) of 0.94 (95% CI 0.87–0.99). Discriminant validity: All PHQ-9 item means were significantly different when comparing non-depressed (CIDI negative) and depressed (CIDI positive) participants Inter-rater reliability: N/A Test–retest reliability: N/A Other: Sensitivity of PHQ-9: For a PHQ-9 score of 10 or greater, the sensitivity was 0.94 (95% CI 0.73–0.99), specificity was 0.80 (95% CI 0.71–0.86), PPV was 0.42 (95% CI 0.27–0.59), and NPV was 0.99 (95% CI 0.93–1.00), with a positive likelihood ratio of 4.62 (95% CI 3.12–6.83), and negative likelihood ratio of 0.07 (95% CI 0.01–0.47). Sensitivity of other idiom/CCD: Heart-mind problems had a sensitivity of 0.94 (95% CI 0.69–1.00), specificity of 0.27 (95% CI 0.19–0.36), PPV of 0.17 (95% CI 0.10–0.26), and NPV of 0.97 (95% CI 0.81–1.00). Brain-mind problems had low sensitivity for CIDI positive status (sensitivity = 0.47, 95% CI 0.25–0.71).
McMullen *et al*. (2012)	**APAI** (depression symptom scale)	Interviewer administered	Internal consistency (*α*): high = 0.93 Validity: N/A Inter-rater reliability: N/A Test–test reliability was strong (*r* = 0.835, *p* < 0.001) Other: N/A
Miller *et al*. (2006)	**Afghan Symptom Checklist (ASCL)**	Interviewer administered	Internal consistency (*α*): High 0.93 Validity: Good construct validity, correlating strongly with a measure of exposure to war related violence and loss (Afghan War Experiences Scale, AWES) (*r* = 70, *p* < 0.001). Inter-rater reliability: N/A Test–retest reliability: N/A The indigenous items were among the most frequently endorsed symptoms of distress on the ASCL.
Mumford *et al*. (2005)	**Pakistani Anxiety and Depression Questionnaire** **(PAD-Q)** (with 2 subscales, ‘AD’ and ‘D’)	Either self-report or interviewer administered	The internal consistency (*α*) of the ‘AD’ scale was 0.92 (95% CI 0.90–0.94) and for the ‘D’ scale was 0.91 (95% CI 0.89–0.93). Validity Discriminant validity: A histogram of ‘AD’ (anxiety/depressive disorders) scale scores showed a clear bimodal distribution between controls and cases. The histogram of ‘D’ scale (depressive disorders) scores was weakly bimodal. Inter-rater reliability: N/A Test–retest reliability: N/A Other: Sensitivity and specificity were all >80% apart from the specificity of the ‘D’ scale in a sample of depressive *v*. anxiety patients
Patel *et al*. (1997)	**Shona Symptom Questionnaire (SSQ)**	Interviewer administered	Internal consistency (*α*) = 0.85 Validity: Discriminant validity: Cases had significantly higher scores than non-cases (mean score, 8.6; 95% CI 7.9–9.2 *v*. mean score, 4.1; 95% CI 3.6–4.5; *p* < 0.001) Divergent validity: Positive Mental Health Items were all significantly more common among non-cases (*r* = −0.54, *p* < 0.001). The total score correlated strongly with patients' self-assessment of the emotional nature of their illness. Inter-rater reliability: N/A Test–retest reliability: N/A Other: Validity coefficients: ROC curves suggested an optimal cut-off point of 7/8 (of 14) (area under curve, 0.88; s.e., 0.02).
Phan *et al*. (2004)	**Phan Vietnamese Psychiatric Scale (PVPS)**	Interviewer administered	Internal consistency (*α*) for the subscales ranged from 0.87 to 0.95 Validity Construct validity: Factor analysis – the proposed four-factor structure of the PVPS appears to represent the best four-factor arrangement of the items Multitrait – multimeasure analysis also supported the construct validity of the scale Of all measures, the PVPS showed the most consistent evidence of discriminant validity The PVPS demonstrated good criterion validity against case assignments by psychiatrists, naturalist healers, and structured diagnostic measures. Inter-rater reliability: N/A Test–retest reliability: Test–retest correlations coefficients were 0.89 for the depression scale (0.88 for affective subscale, 0.89 for the psychovegetative subscale); 0.81 for the anxiety scale, and 0.84 for the somatisation scale. Other: The PVPS was rated by patients as more acceptable in comparison with other related measures. A larger proportion of patients assessed the PVPS as being more culturally sensitive than other measures.
Rasmussen *et al*. (2014)	**ASCL**	Interviewer administered	Internal consistency (*α*) of the subscales was as follows: Fisher = −0.23 (unstable); Jigar Khun = 0.91; Aggression = 0.66 (both satisfactory) Validity Construct validity of the scale was carried out by comparing the items' face validity and using exploratory and confirmatory factor analysis: The 3-factor model suggested by the EFA fit the two confirmatory samples adequately (CFI = 0.943, TLI = 0.974, RMSEA = 0.087 in confirmatory sample 1; CFI = 0.920, TLI = 0.959, RMSEA = 0.099 in confirmatory sample) Only the second two subscales were used for determining the external validity by association with traumatic exposure and wealth indices. Trauma exposure and wealth were significantly correlated across subscales for women (trauma exposure: Jigar Khun = 0.28, Aggression = 0.25; wealth: Jigar Khun = −0.28, Aggression = −0.27), but inconsistently so for men (trauma exposure: Jigar Khun = 0.18, Aggression = 0.01; wealth: Jigar Khun = −0.11, Aggression = 0.06). This suggests that external validity of the scale was gender dependent. Inter-rater reliability: N/A Test–retest reliability: N/A The ASCL was a better measure of distress than the SRQ-20 for women, while the two measures were similar for men.
Rasmussen *et al*. (2015)	**Zanmi Lasante Depression Symptom Inventory (ZLDSI)**	Interviewer administered	Internal consistency: N/A Validity Discriminant validity: Depressed participants (*M* = 21.43, s.d. = 8.31) scored statistically significantly higher than not depressed participants (*M* = 14.05, s.d. = 9.60; *t*_(103)_ = 4.17, *p* < 0.001), with a large effect (Cohen's *d* = 0.82). Convergent validity: Total scores were strongly associated with functional impairment (WHODAS-II scores), r = 0.71 (*p* < 0.001). Inter-rater reliability: N/A Test–retest reliability: N/A Other: ROC analysis predicting clinical diagnoses from total scores suggested moderate predictive accuracy. The AUC was 0.71, 95% CI 0.61–0.81. The scale had acceptable sensitivity but did less well with specificity which could not be improved without unacceptable losses in sensitivity
Roberts *et al*. (2006)	**Hwa-Byung (HB) scale**	Self-report	Internal Consistency: N/A Validity Convergent validity: Correlation with the somatic MMPI-2 scale: *H*_s_, which measures somatic complaints (0.75), *D*, which measures symptoms of depression including feelings of sadness (0.52), pessimism, and psychomotor retardation and *H*_y_, which measures the development of physical symptoms in response to stress (0.47). The MMPI-2 psychological and clinical content scales that were hypothesised to correlate best with HB (i.e. HEA, ANX and DEP) yielded correlations of 0.60 or greater. Correlation between the HB scale and the Peer Rating Form (developed by the authors to serve as a measure of external validation by identifying 13 peer-rating items that appeared to address the somatic and psychiatric symptoms associated with HB) was moderate (0.21) Inter-rater reliability: N/A Test–retest reliability: N/A Other: N/A
Silove *et al*. (2009)	**Index of explosive anger**	Interviewer administered	Internal consistency: N/A Construct validity: Theoretically driven predictors were associated with explosive anger, such as exposure to past trauma events where there was an increase in odds according to the number of events endorsed, with a major increase for the highest trauma endorsement group (odds, 95% CI): for 6–10 trauma categories; 3.4 (1.6–7.0); for 11–15 categories: 4.9 (2.2–10.8); and for 16+ categories: 10.7 (4.1–27.3) (Wald: 45.58, *p* = 0.000). Inter-rater reliability: N/A Test–retest reliability: N/A Other: N/A
Snodgrass *et al*. (2017)	**Positive and Negative Affect Scale (PANAS)**	Interviewer administered	Internal consistency: *α* = 0.87 Validity: (Note: higher score indicates more positive emotions and less negative emotions unless otherwise indicated) Convergent validity: Correlation with Hopkins Symptom Checklist (HSCL-10), *r* = −0.49; Bradford Somatic Index (BSI), *r* = −0.34; Correlation with Physical Illness Scale, *r* = −0.30; 4-item stress scale, *r* = −0.50; 4-item subjective wellbeing, *r* = 0.63 Construct validity: Associated as expected with gender, education, income, and household size; Mean scores for *negative* emotion scale were greater in villages exposed to greater stressors (e.g. relocation, deforestation). Content validity: Items loaded onto factors in a way that matched ethnographic data and literature Inter-rater reliability: N/A Test–retest reliability: N/A Other: N/A
Weaver (2017)	**Tension scale**	Interviewer administered	Internal consistency: Validity: Convergent validity: correlation with HSCL-25 *r* = 0.778, *p* < 0.01 for women without diabetes; *r* = 0.807, *p* < 0.01 for women with diabetes; HSCL-25 depression and anxiety components *r* = all above 0.7, *p* < 0.01. Discriminant validity: those who endorsed no experience of a ‘tension’ scale item scored significantly lower on the HSCL Inter-rater reliability: N/A Test–retest reliability: N/A Other: N/A
Weaver & Hadley (2011)	**Tension scale**	Interviewer administered	Internal consistency: *α* = 0.93 Validity: Construct validity: Factor analysis revealed one dominant factor in the ‘tension’ scale Convergent validity: HSCL and ‘tension’ scores were moderately correlated (*r* = 0.56, *p* < 0.01) Inter-rater reliability: N/A Test–retest reliability: N/A Other: N/A

##### Interventions

In the two studies that examined an intervention that incorporated the use of idioms/CCDs, the intervention produced significantly better outcomes than the control conditions (Choi & Lee, 2007; Hinton *et al*., 2011); however, determining whether the incorporation of idioms/CCDs was a factor in the effectiveness of the intervention was not a specific aim of the studies, so we are not able to evaluate what intervention effects are due to idioms/CCDs. The study by Hinton *et al*. (2011) was a pilot study comparing an intervention that incorporated the use of idioms/CCDs into a culturally adapted CBT (CA-CBT) for Latina women with treatment-resistant PTSD and compared this intervention with applied muscle relaxation. No information about the perceived acceptability of the intervention was provided in the study. The study by Choi & Lee (2007) integrated information about the idiom/CCDs into a culturally tailored nursing programme. The study did not discuss initial piloting or recipients' perceptions of the acceptability of the intervention.

## Discussion

The current review gathers together, for the first time, research papers that discuss the integration of idioms/CCDs into psychological/psychosocial assessment measures and interventions. The review aimed to synthesise information on how idiom/CCD data were gathered, how their integration occurred, whether appropriate translation procedures were adhered to and the acceptability of these assessment measures/interventions.

In terms of the methods used to identify idioms/CCDs (review question 1), primary qualitative research was the most commonly used method. The justification for the choice of study language varied across the studies. In two studies, research was carried out in the preferred language of participants (Weaver & Hadley, 2011; Rasmussen *et al*., 2014); in three studies it was carried out in the official language of the country where research was undertaken (Hinton *et al*., 2011; Kaiser *et al*., 2013, 2015) and all other studies chose one language from a range of languages spoken in a setting where the research was conducted. This monolingual approach to working can belie the multilingual realities of people's daily lives where a number of languages can be in play (Andrews *et al*., 2018). The practicalities of research mean that linguistic diversity can be difficult to accommodate; however, the marginalisation of particular languages and the risks that this may pose for certain communities needs to be recognised.

Regarding the integration of idioms/CCDs into assessments and interventions (review question 2), new assessment measures were developed by creating items based on signs and symptoms identified in qualitative interviews. For studies that created local assessment measures for constructs that had been identified elsewhere like depression or anger attacks, they either created new scales (Mumford *et al*., 2005; Abeyasinghe *et al*., 2012); adapted a pre-existing scale using the locally identified symptoms (Silove *et al*., 2009; Kohrt *et al*., 2016); or used a combination of pre-existing and new scales (Rasmussen *et al*., 2015). In terms of interventions, idioms/CCDs were incorporated into CBT (Hinton *et al*., 2011) or a mix of music therapy, drama and group therapy (Choi & Lee, 2007).

Regarding translation processes (review question 3), some papers did not sufficiently discuss translation, with six studies failing to detail translation processes of idiom/CCD terms (Mumford *et al*., 2005; Roberts *et al*., 2006; Choi & Lee, 2007; Silove *et al*., 2009; Hinton *et al*., 2011), perhaps due to the fact that translation only occurred after the research process was completed for the purposes of publishing the paper in an academic journal. Of the studies that did describe translation processes, all studies except for one (Miller *et al*., 2006) were graded ‘unclear’ or ‘inadequately described’ for at least one question out of eight in the COSMIN assessment. Twelve studies in this review compared idioms/CCDs with psychiatric constructs in some way. White *et al*. (in press) have raised concerns about the risk of epistemic injustice occurring when indigenous knowledge is subsumed into constructs developed in the Global North^2^. It is also important that translation of information gathered on idioms/CCDs is conducted in a systematic and reflective manner, even if the translation only occurs after the research has been carried out for the purposes of the paper. With this in mind, White *et al*. (in press) have constructed a Group Reflection Tool that can be used following communications about distress and/or wellbeing that have involved an interpreter. As one example of these types of comparisons, Abeyasinghe *et al*. (2012) created a symptom scale of locally identified idioms of distress and other ‘universal terms’ that correlated with the diagnosis of depression in the DSM. They concluded that, ‘only certain idioms of distress are useful in detecting pathological states such as depression’ (p. 147). In such circumstances, it is important to be aware of the risk of *category fallacy* (Kleinman, 1977) as the authors are noting associations between local idioms and the depression construct and subsequently describing those local idioms as symptoms of depression. This neglects the possibility that locally identified idioms may be useful in detecting culturally specific pathological states. On the other hand, Fernando (2008) appeared to deal with translation and sensitivity to semantic equivalence well. The *emic-etic* dilemma experienced by the author in attempting to give English language terms to the factors of the scale was discussed, and it was noted that certain terms could not easily be translated into diagnoses developed in the Global North without losing meaning.

With regard to review questions 4 and 5, all studies assessed the participants' level of understanding of the measures incorporating idioms/CCDs either during testing or through prior piloting. The majority of studies reported that these assessment measures had good psychometric properties. The most commonly reported psychometric property was internal consistency, with all of these assessment measures showing good or high internal consistency. A range of different approaches was used across the studies to determine the validity of the measures that incorporated idioms/CCDs, and the majority was shown to have moderate to high levels of validity. One study (Phan *et al*., 2004) gathered participants' views on the cultural sensitivity of a measure that incorporates idioms/CCDs, in comparison with standard measures. This is an important gauge of the acceptability of a measure, and researchers should explicitly gather this information in future. It is important to note however that this review does not cover all of the studies that have carried out psychometric testing of assessment measures that included idioms/CCDs, as only studies that reported on development and testing of scales were included.

None of the studies included in this review reported on efforts to incorporate idioms/CCDs into assessment measures/interventions that were intended for majority populations living in the Global North. This conveys a potential lack of parity in mental health research where *idiomatic blind-spots* (i.e. an obscuring of the culturally situated nature of one's own distress) may be occurring in the Global North. This is consistent with the previously noted observation that none of the CCDs listed in the glossary of the DSM-5 are English language terms, and none are deemed to originate in North America or Europe. These descriptions of idioms/CCDs do not form part of the taxonomy of mental illness in the main text but are instead restricted to the appendix (Thornton, 2017). Consistent with views previously expressed by commentators (Kleinman, 1977), it seems that there is an assumption that the largely English language terminology used to describe ‘mental disorders’ in the Global North are capturing experiences that are not culturally constructed but are instead universal forms of *mental illnesses* that are not prone to cultural particularity. This can create circumstances where non-English language terms for distress are bench-marked against, or indeed subsumed by, psychiatric terminology on the grounds that the latter carries greater legitimacy. This is a particular risk when local language terminology is translated into English and ‘conceptual equivalence’ is deployed (see White *et al*., in press). It is thus important to raise awareness among mental health practitioners and researchers of the potential benefits of sharing knowledge about perceptions of distress and/or wellbeing in diverse linguistic and cultural settings, and of avoiding the imposition of concepts that are commonplace in majority languages like English.

The majority of included studies addressed assessment measures. However, the two studies that integrated idioms/CCDs into interventions produced better outcomes when incorporating idioms into an intervention compared with the standard intervention. Thus there is potential for future research to further explore the use of idioms/CCDs in mental health interventions. A number of approaches have been proposed for adapting and translating interventions and assessments for local contexts, e.g. the Design, Implementation, Monitoring and Evaluation (DIME) (Applied Mental Health Research Group, 2013), a ‘culturally sensitive framework’ (Bernal & Sáez-Santiago, 2006) and the translation monitoring form (Van Ommeren *et al*., 1999).

### 

#### Limitations

The current review had a number of limitations. First, the terminology used to refer to idioms/CCDs varied greatly between papers and included many different terms. This is similar to what Kohrt *et al*. (2014) found in their systematic review of CCDs – that the terminology used to refer to locally distinct ways of expressing distress (idioms, CCDs etc.) was not uniform across papers or research teams. The wide variety of terms and phrases used may be one reason that a substantial number of papers included in this review were found from searching reference lists, as it is possible that the search terms were not exhaustive enough. This is a difficulty inherent in conducting a review in an evolving area of study and practice: the concept being explored in this review is relatively new, and no papers before 1997 met the inclusion criteria. Moving forward, it will be important to ensure that researchers investigating linguistic expressions of distress that are specific to particular localities and cultural groups use the terminology ‘idioms/CCDs’ to refer to these phenomena.

The authors recognise the important role of ethnographic research methods for examining the utility and validity of idioms/CCDs in assessments and interventions. However it was beyond the scope of the current review to conduct a thorough examination of the ethnographic methods employed across the studies, which is a potential limitation of this review. A further potential limitation of the current review is that it included only published articles and the included papers were selected according to the parameters of our inclusion criteria. Thus, many studies relevant to this topic of idioms/CCDs were not included.

A final limitation relates to the fact that the scope of the current review was specifically limited to the linguistic translation of narrative descriptions of idioms/CCDs. A key focus was the attention allocated to processes of translation when integrating idioms/CCDs into assessment measures and interventions, however it should be noted that cross cultural mental health related work may well involve working with idioms that are ‘untranslatable’ (Kirmayer & Swartz, 2013). In other words, irrespective of how much care and attention is allocated to processes of forward- and backward-translation, the sharing of concepts relating to mental health across languages may continue to prove challenging. There is a need for further research, and synthesis of this research, relating to: (1) the methods used to collect information about idioms/CCDs – including ethnographic research methods; (2) how the idioms/CCDs were contextualised in terms of local meaning – including how consistent the operationalisation of the idioms/CCDs was with local ethnopsychology and (3) reflections from stakeholders about the clinical utility of the idioms/CCDs in the assessed studies.

## Conclusion

We advocate for the integration of idioms/CCDs into assessment measures and interventions to increase understanding of forms of suffering, improve clinical communication and treatment outcomes, and reduce stigma (Kaiser *et al*., 2015). The studies included in the review demonstrate the efforts that some researchers have made to integrate local ways of understanding and expressing distress into GMH assessment measures and interventions. A key finding was that a large proportion of studies did not engage in particularly rigorous forward- and backward-linguistic translation procedures. In addition, there was a tendency for idioms/CCDs to be integrated into existing assessment measures that had been developed in the Global North. Further research is needed reporting on the *emic*/*bottom-up* development of assessment measures developed in local contexts of the Global South. This is in keeping with calls for more equitable exchanges of knowledge about mental health and wellbeing between the Global North and the Global South (White *et al*., 2014).

## Footnotes

† The notes appear after the main text.

1 Terminology for validity tests differed across publications; in Table 2, we use authors' terminology.

2 The classification between ‘Global North’ and ‘Global South’ is derived from the Brandt Line that was introduced in the 1980s to illustrate the geographic division between more (i.e. Global North), and less (i.e. Global South), economically developed countries across the globe. Although this approach has a number of limitations, the opportunities it facilitates for reflecting on how resources are distributed across the globe are helpful for the purposes of this paper.
